# Whole genome analysis of Black Bengal goat from Savar Goat Farm, Bangladesh

**DOI:** 10.1186/s13104-019-4700-7

**Published:** 2019-10-24

**Authors:** Shah Md. Ziqrul Haq Chowdhury, K. H. M. Nazmul Hussain Nazir, Saam Hasan, Ajran Kabir, Md. Muket Mahmud, Mahdi Robbani, Tahmina Tabassum, Tamanna Afroze, Aura Rahman, Md. Rafiqul Islam, Maqsud Hossain

**Affiliations:** 1 Livestock Division, Bangladesh Agricultural Research Council, Farmgate, Dhaka, Bangladesh; 2 Department of Microbiology and Hygiene, Faculty of Veterinary Science, Bangladesh Agricultural University, Mymensingh, 2202 Bangladesh; 3 Department of Biochemistry and Microbiology, NSU Genome Research Institute (NGRI), Baridhara, Bashundhara, North South University, Dhaka, 1229 Bangladesh; 4 NSU Genome Research Institute (NGRI), Baridhara, Bashundhara, North South University, Dhaka, 1229 Bangladesh

**Keywords:** *Capra hircus*, Bangladesh, Black Bengal goat

## Abstract

**Objectives:**

Single nucleotide polymorphisms (SNPs) play critical roles in genetic diversity and disease. Many traits and diseases are linked with exonic SNPs that are significant for gene function, regulation or translation. This study focuses on SNPs that potentially act as the genetic basis for desirable traits in the Black Bengal Goat. This variety of goat is native to South Asia, and is identified as one of the most commercially important meat producing animals in the world. The aim of this study was to sequence the genome of Black Bengal Goats and identify SNPs that might play a significant role in determining meat quality in the organism. The study focuses on exonic SNPs for their greater likelihood of affecting the final translated protein product.

**Results:**

Approximately 76,000 exonic variants were identified in the study. After filtration using a Wilcoxon test based score, the number came down to 49, 965 which were found to be distributed in 11,568 genes. The functional pathways affected by these variations included fatty acid metabolism and degradation, which are important processes that influence meat quality.

## Introduction

The Black Bengal goat (*Capra hircus*) is a regional breed found in Bangladesh and eastern India. It is characterized by its smaller stature and is preferred in the livestock industry for higher reproductive rate and quality of meat and skin [1]. Meat quality in goats is measured by several indicators, the most important of which is the degree of tenderness. Previous studies have investigated the correlation between tenderization of meat and the expression of various genes [2]. The goal of this study was to identify specific genetic markers that may correlate to improved meat quality observed in the Black Bengal goat using a whole genome sequencing (WGS) approach. Variant calling and single nucleotide polymorphism (SNP) profiling was carried out on the sequenced data through comparison with previously shortlisted key genes so as to identify those variants that may affect these candidate genes and possibly confer changes that lead to desirable characteristics in the organism. The variants were also annotated to determine their downstream effect on transcriptional and translational processes. The rationale of this study was to identify SNPs, either unique changes or differences in the number of such variants, in various genes that may help in better understanding and characterizing the factors that determine meat quality in goats.

## Main text

### Methods

Blood sample from a female Black Bengal goat aged 4 years was collected from Savar Goat Farm. The blood samples were transported to the Department of Microbiology and Hygiene, Bangladesh Agricultural University (BAU) for DNA extraction and sample processing. All goats were handled following standard guidelines of Animal Welfare and Experimental Ethical Committee (AWEEC) of BAU [approval number AWEEC/BAU/2019 (13)].

DNA from the blood sample was extracted using QIAamp^®^ DNA Blood Mini kit (QIAGEN, Germany) following manufacturer’s instructions. DNA purity was evaluated by NanoDrop 1000 Spectrophotometer (Life Technologies, CA, USA) and 0.8% agarose gel electrophoresis. Sequencing was carried out at the Genewiz Sequencing Centre in Suzhou, China using an Illumina HiSeq platform that produced 1,151,332 trimmed reads (phred quality > 30) and a 109-fold mean coverage.

For genome mapping ARS1 (accession no. GCA_001704415.1) was used as the reference genome and the MEM algorithm of Burrows–Wheeler Aligner (BWA) [3] was selected with the minimum mapping quality set to 20 and unmapped reads were filtered out using SAMtools [4]. Following this, read groups were added and PCR duplicates were marked using Picard (http://broadinstitute.github.io/picard/). For SNP profiling and variant calling, a modified version of the Genome Analysis Toolkit (GATK) best practices pipeline was followed (DePristo et al. [9]), Base recalibration was performed with BaseRecalibrator, and finally variants were called with HaplotypeCaller in Genomic Variant Call Format (GVCF) mode. Variants were further analysed on HaplotypeCaller based on several statistical parameters including QualByDepth (QD), Wilcoxon test scores and fisher test score for strand bias.

The variants were annotated with ANNOVAR (ANNOate VARiation) [5], with an inbuilt annotation database using gene annotation data from UCSC (University of Santa Cruz) Genome Browser [6]. In the next step, gene variants that have been previously implicated in meat tenderization were analyzed. Variants which passed the BaseQRankSumTest filter, a Mann–Whitney-Wilcoxon U-test were considered for further analysis. Filtration was carried out by the ReadPosRankSum values which are calculated based on the result of the BaseQRankSumTest for each variant. The Manhattan plot was generated using qqman package available on R [7]. Further functional annotation and pathway analysis of the final RNA editing sites was carried using the Database for Annotation, Visualization and Integrated Discovery (DAVID) [8].

### Results

Genome variants belonging only to the exonic regions as per ANNOVAR’s algorithm [5], were considered to look into the functional regions of the genome. Exonic variants are more likely to affect the final protein product, possibly through alterations of gene function, expression, translation or even post-translational modifications. Following this filtration, 76,000 exonic variants were found and the number was reduced further to 49,965 after a second filtration based on Wilcoxon test scores as calculated by the BaseQSum calculator functionality of GATK’s variant annotator [9].

These 49,965 exonic variant sites were distributed in a total of 11,568 genes. These variants were categorized according to their effect on translation, i.e. synonymous, non-synonymous, stop-gain, frame-shift or unknown. To statistically analyze the association study, the u-based z approximation derived from the rank-sum test was used instead of the more conventional p-values.

**Fig. 1:**
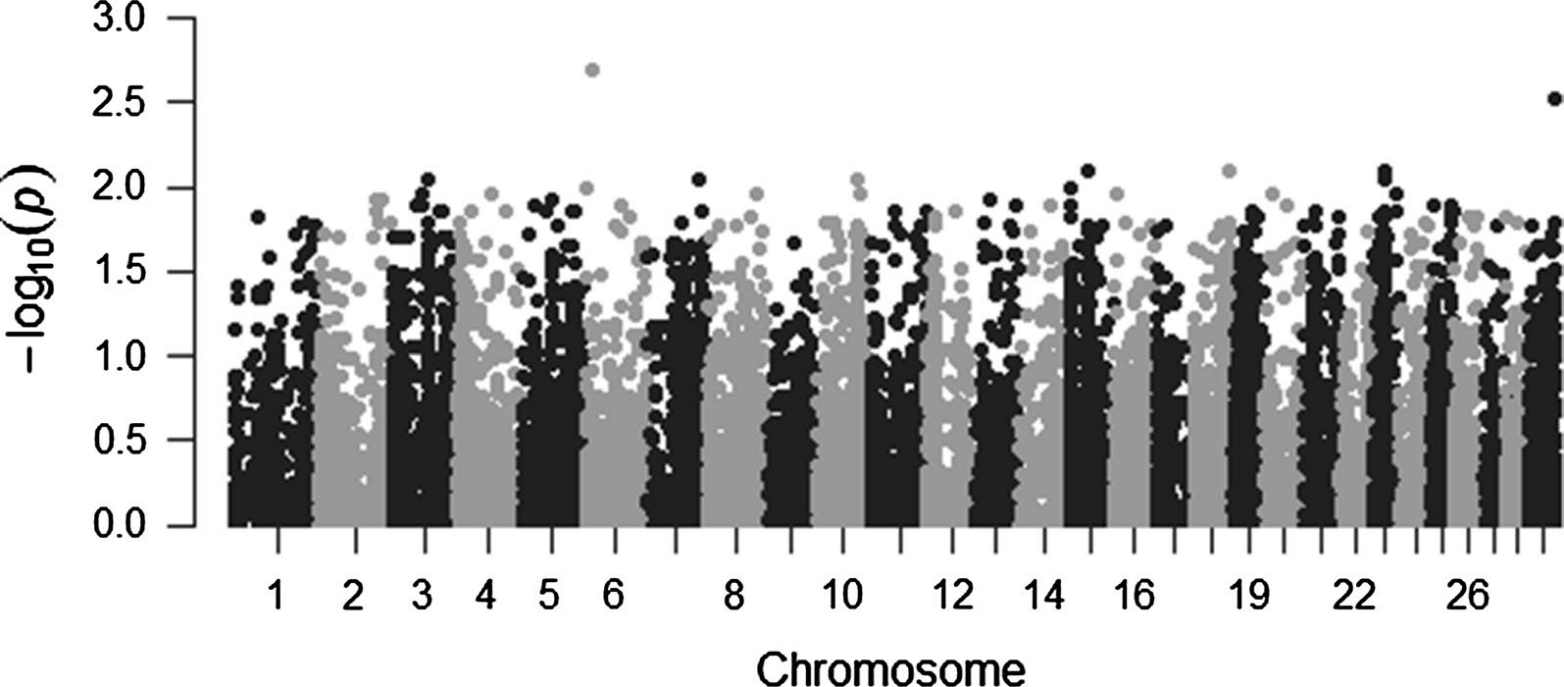
Manhattan plot of U-based Z approximation scores of SNPs per chromosome. The graph shows SNPs and their associated ReadPosRankSum values for each chromosome

GATK’s variant annotation functionalities were further used to calculate these values which are outputted as the ReadPosRankSum values. Negative values were omitted owing to those representing presence of the alternate alleles near the ends of reads which are often erroneous. The positive values were used to plot the frequency of variants per chromosome and used to speculate on possible chromosome-wise association between the variants and possible desired traits (Fig. 1).

Previous studies have shown the association of certain gene products with the meat tenderization process, including calpain-1 (CAPN1), calpain-2 (CAPN2), calpastatin (CAST), caspase 3 (CASP3), caspase 9 (CASP9), αB-crystallin (CRYAB), heat shock protein 27 (Hsp27), heat shock protein 40 (Hsp40) and heat shock protein 70 (Hsp70) (Saccà et al., 2019). Among these, this study found variants in Hsp70, CASP3, CAPN1, CAPN2 and CAST encoding genes. Table 1 shows the positions and types of variants found in these genes.

**Table 1:** Variants found in genes previously implicated in meat tenderization process

*Gene*	Chromosome	Position	Base change	Amino acid change
*CAST*	NC_030814	14434086:exon3:c	G83A:p	R28 > K
*CAST*	NC_030814	14434086:exon18:c	C1245G:p	V415 > V
*CAST*	NC_030814	14434086:exon28:c	G2052A:p	E684 > E
*CAST*	NC_030814	14434086:exon25:c	C1863T:p	P621 > P
*CAST*	NC_030814	14434086:exon14:c	G972A:p	P324 > P
*Hsp70.1*	NC_030830	22440674:exon1:c	G309A:p	V103 > V
*Hsp70.1*	NC_030830	22440674:exon1:c	C993T:p	I331 > I
*Hsp70.1*	NC_030830	22440674:exon1:c	C1653T:p	S551 > S
*Hsp70.1*	NC_030830	22440674:exon1:c	C1528T:p	L510 > L
*Hsp70.1*	NC_030830	22440674:exon1:c	C24A:p	G8 > G
*CAPN1*	NC_030836	43822608:exon17:c	C1803A:p	R601 > R
*CAPN2*	NC_030823	25579185:exon12:c	G1522A:p	D508 > N
*CAPN2*	NC_030823	25579185:exon13:c	T1560A:p	L520 > L
*CAPN2*	NC_030823	25579185:exon17:c	G1764A:p	G588 > G
*CAPN2*	NC_030823	25579185:exon18:c	C1833T:p	Y611 > Y
*CAPN2*	NC_030823	25579185:exon3:c	C327T:p	A109 > A
*CASP3*	NC_030834	30502447:exon3:c	C267T:p	N89 > N

The diversity among nonsynonymous substitutions is significantly lower than among synonymous substitutions since they are subject to higher selective pressures [10]. Further analysis of non-synonymous variants affecting the encoded proteins revealed a total of 20,879 non-synonymous variants that were distributed in 6850 genes. The gene ontology analysis provided some promising findings. In particular, the functional clustering analysis showed that the processes affected by the identified genes were fatty acid metabolism and fatty acid degradation. Both of these processes may hold significant contributions to the leanness and eventual quality of meat produced from the concerned specimen [11]. The third pathway with the most significant scores from the list of genes was the PPAR pathway (peroxisome proliferative-activated receptor) which also plays role in fatty acid metabolism. Table 2 shows these results alongside the associated statistical scores as calculated by DAVID’s in-built algorithm.

**Table 2:** Pathways affected by the identified SNPs and their associated statistical scores

Annotation cluster 1	Enrichment score: 3.16	Count	*p* value	Benjamini
KEGG_PATHWAY	Fatty acid degradation	17	3.40E−06	1.80E−04
KEGG_PATHWAY	Fatty acid metabolism	15	1.00E−03	9.70E−03
KEGG_PATHWAY	PPAR signalling pathway	13	9.20E−02	3.40E−01

Annotation cluster 2	Enrichment score: 2.8	Count	p-value	Benjamini
KEGG_PATHWAY	Protein digestion and absorption	36	2.90E−08	3.80E−06
KEGG_PATHWAY	ECM-receptor interaction	22	5.20E−04	5.50E−03
KEGG_PATHWAY	Focal adhesion	34	2.30E−02	1.20E−01
KEGG_PATHWAY	Amoebiasis	20	3.10E−02	1.50E−01
KEGG_PATHWAY	PI3K-Akt signaling pathway	32	9.40E−01	9.90E−01

### Discussion

The main objective of the study was to systematically identify genomic variants underlying major phenotypic traits in Black Bengal goat obtained via whole genome-sequencing data.

A total of 11,564 genes were identified from a list of 47,241 exonic variants. Among these a total of 20,879 non-synonymous mutations were found in 6850 genes. The search for exonic variants were narrowed down to non-synonymous sites since they exert direct effect on the translated protein sequence and thus often mark polymorphisms that contribute to greater fitness and virulence potentials in the organism.

This study provides evidence regarding the significance of certain gene variants in influencing beneficial characteristics of the Black Bengal goat. Among these variants, two contained non-synonymous changes that led to alterations in the encoded amino acid. CAST contained a variant that would lead to an arginine being substituted for lysine at position 28. CAPN2 contained a change that would result in an aspartic acid being substituted by asparagine at position 508. In addition several variants in HSP70 protein were also found, along with considerable numbers of more in other HSP proteins. The HSP family has previously been heavily linked with the process of meat tenderization [2]. The lipid metabolism pathways being highlighted by the ontology analysis is another indication of specific variants to the observable characteristics of these organisms [11].

In conclusion, we would like to report this study as a preliminary insight into the possible genetic basis of improved meat quality in Black Bengal goats. The goat remains an important organism in the livestock industry and hence its understanding and improvement hold significant economic value. Further genomic characterization and analysis of this breed of goat with substantially larger sample size would be able to verify our initial findings and add new evidence that would allow a better discerning of the unique variants that might confer this species its desirable traits.

## Limitations

In a number of previous studies association with particular gene/s and trait has been investigated and we investigated the association between such known genes and particular trait of Black Bengal Goat such as meat tenderization process using only one sample as carried out in previous studies [12, 13].

We have also carried out de novo identification of genes and their ontology although looking into more genomes might have provided greater insight and more statistical power. Although only one sample was used we took rigorous statistical approach to filter out spurious variants. However, this initial finding is also extremely important looking into the cost of sequencing technologies and in the context of a developing country like Bangladesh. Certainly further comparative genomics analysis using a larger number samples will be carried out based on these initial findings.

De novo genome assembly was not carried out due to lack of enough computational power in terms of RAM (random accessory memory) and this could have provided information on the identification of gene gain/loss compared to other goat genomes available in genome databases.

This is also to be noted that the outcome of this particular study using only one genome will certainly provide confidence to the scientific community in this region for in detailed genomics analysis of large eukaryotic genomes and facilitate further genomic research in resource poor settings like Bangladesh.

## Data Availability

The sequenced genome has been submitted to NCBI and the data has been made publicly available (SRA ID: SRR8928054, BioProject ID: PRJNA53351, BioSample ID: SAMN11458493).

## Abbreviations

ANNOVAR: ANNOtate VARiation
AWEEC: Animal Welfare and Experimental Ethical Committee
BAU: Bangladesh Agricultural University
BARC: Bangladesh Agricultural Research Council
BWA: Burrows–Wheeler Aligner
DAVID: Database for Annotation, Visualization and Integrated Discovery
GATK: Genome Analysis Toolkit
GVCF: Genomic Variant Call Format
PPAR: peroxisome proliferative-activated receptor
QD: QualByDepth
RAM: random accessory memory
SNP: single nucleotide polymorphism
UCSC: University of Santa Cruz
WGS: whole genome sequencing
